# Monte Carlo simulation for scanning technique with scattering foil free electron beam: A proof of concept study

**DOI:** 10.1371/journal.pone.0177380

**Published:** 2017-05-11

**Authors:** Wonmo Sung, Jong In Park, Jung-in Kim, Joel Carlson, Sung-Joon Ye, Jong Min Park

**Affiliations:** 1 Program in Biomedical Radiation Sciences, Department of Transdisciplinary Studies, Seoul National University Graduate School of Convergence Science and Technology, Seoul, Republic of Korea; 2 Department of Radiation Oncology, Seoul National University Hospital, Seoul, Republic of Korea; 3 Institute of Radiation Medicine, Seoul National University Medical Research Center, Seoul, Republic of Korea; 4 Biomedical Research Institute, Seoul National University Hospital, Seoul, Republic of Korea; 5 Institute for Smart System, Robotics Research Laboratory for Extreme Environments, Advanced Institutes of Convergence Technology, Suwon, Republic of Korea; Tsinghua University, CHINA

## Abstract

This study investigated the potential of a newly proposed scattering foil free (SFF) electron beam scanning technique for the treatment of skin cancer on the irregular patient surfaces using Monte Carlo (MC) simulation. After benchmarking of the MC simulations, we removed the scattering foil to generate SFF electron beams. Cylindrical and spherical phantoms with 1 cm boluses were generated and the target volume was defined from the surface to 5 mm depth. The SFF scanning technique with 6 MeV electrons was simulated using those phantoms. For comparison, volumetric modulated arc therapy (VMAT) plans were also generated with two full arcs and 6 MV photon beams. When the scanning resolution resulted in a larger separation between beams than the field size, the plan qualities were worsened. In the cylindrical phantom with a radius of 10 cm, the conformity indices, homogeneity indices and body mean doses of the SFF plans (scanning resolution = 1°) vs. VMAT plans were 1.04 vs. 1.54, 1.10 vs. 1.12 and 5 Gy vs. 14 Gy, respectively. Those of the spherical phantom were 1.04 vs. 1.83, 1.08 vs. 1.09 and 7 Gy vs. 26 Gy, respectively. The proposed SFF plans showed superior dose distributions compared to the VMAT plans.

## Introduction

Advanced radiotherapy techniques such as intensity modulated radiation therapy (IMRT) as well as volumetric modulated arc therapy (VMAT) enable conformal delivery of a prescription dose to deeply seated target volumes while sparing dose to normal tissues [1–4]. However, in the case of tumors located at shallow depths, photon-based radiotherapy techniques are sometimes problematic even with IMRT and VMAT due to the absence of electronic equilibrium at the patient surface, as well as the deep penetration of photon beams, which may deliver unwanted doses to deeply seated organs at risk (OARs) [5]. Therefore, high-energy electron beams with a bolus are generally used for the treatment of shallow tumors as they have a relatively low dose range which prevents irradiation of the deeper parts of a patient body [5].

Electron beam therapy is the best treatment option for shallow tumors located near flat surfaces of a patient’s body. However, if the patient surface is irregular, such as the scalp or foot, the penetrating power of an electron beam is modified in proportion to the off-axis distance, which results in heterogeneous delivery of the dose to a shallow target volume, such as those for skin cancer [3, 5, 6]. Therefore, modulated electron radiation therapy (MERT) has been suggested by several studies [7–12]. By modulating the electron beam intensity, as well as the energy, these studies have demonstrated that an optimal dose distribution could be delivered to a patient with shallow tumors near an irregular surface. To modulate electron beams, various groups have suggested add-on electron multi-leaf collimators (MLCs) located close to the patient body, while others have suggested modifying the treatment head and using the existing photon MLC [7–12]. However, one issue with using the photon MLC is the bremsstrahlung photon contamination that accompanies the primary electron beam [7]. To overcome this problem, Connell *et al*. suggested using scattering foil free (SFF) electron beams for MERT [7]. They investigated the characteristics of SFF electron beams with Monte Carlo (MC) simulations and showed an increased dose rate as well as decreased bremsstrahlung photon contamination. Eldib *et al*. investigated the clinical potential of SFF electron beams [13]. They demonstrated clinically acceptable dose distributions with SFF electron beams for the conventional electron boost of the breast tumor bed. On the other hand, Wu *et al*. suggested a new concept: dynamic electron arc radiotherapy (DEAR), which utilized modulation in gantry rotation, dose rate and couch motion simultaneously [14]. They showed superior dose distributions in a cylindrical phantom as compared to conventional electron treatment.

The one of problems of MERT is that stopped beam delivery, which arise increased delivery time and dose uncertainties, when changing electron beam energy during treatment [11]. In addition, MERT is not appropriate for a large tumor such as skin cancer on the scalp. Similar to MERT, DEAR is also inappropriate for treating skin cancer located on the scalp, as the human head is spherically-shaped rather than cylindrical [14]. For the treatment of extensive or multi-focal skin cancer developed on irregular surfaces, several studies have demonstrated that superior dose distributions can be acquired with the VMAT technique as compared to conventional electron radiotherapy, 3D conformal radiation therapy (3D CRT) and high-dose-rate (HDR) brachytherapy with applicators [3, 6]. However, considerable irradiation of normal tissue was still not avoidable even with VMAT since VMAT is a photon-based treatment technique. Currently, these treatment options such as MERT and VMAT are limited for extensive skin cancer on scalp. To address this, we used a scanning technique with SFF electron beams using the magnet in the treatment head. Clinical linacs with scanning beams using the magnet in the treatment head was already introduced clinically as the MM50 racetrack microtron (Scanditronix Medical AB, Uppsala, Sweden) [15]. This machine produces a flat photon beam by scanning of incident electron beams rather than by using a flattening filter [16, 17]. In addition, synchronized operation of treatment couch movement, gantry rotation and monitor unit (MU) delivery is also currently available (ex. developer mode of TrueBeam^™^, Varian Medical Systems, Palo Alto, CA, USA) [14, 18]. If it is possible to combine the magnet in the linac treatment head with synchronized movement of the couch and gantry, it seems feasible to deliver electron beams with a small field that are always perpendicular to the patient surface, while keeping a constant source to surface distance (SSD). Scanning with small-field electron beams perpendicular to the patient surface may be able to deliver a uniform dose to the extensive irregular surface regions without changing electron energies. To shorten treatment time as well as to reduce photon contamination, SFF electron beams would be beneficial for this treatment technique since it is not necessary to deliver a flat electron beam for scanning. Before suggesting SFF electron beam scanning as a clinically viable technique, the dosimetric benefit of this technique should be identified in comparison with conventional treatment techniques. Therefore, as a proof of concept study, we investigated SFF electron beam characteristics with MC simulations and calculated dose distributions in virtual phantoms with the simulated SFF scanning electron beams. For both cylindrical and spherical phantoms of various radii, we compared dose distributions of the SFF scanning electron beam vs. those of VMAT, which is the current state of the art for skin cancer on irregular patient surfaces.

## Materials and methods

### Monte Carlo simulations

In this study, the BEAMnrc MC code was used for the simulation of a clinical linac with and without scattering foil [19]. MC uses basic physics interaction probabilities sampled via selection of random numbers to determine radiation transport. The statistically acceptable results are calculated with a sufficient number of particle transportation. MC is especially useful to predict the potentials of new techniques. For these reasons, a number of general purpose MC codes are publicly available. Among them, the BEAMnrc has been thoroughly established as being reliable and accurate tools, specifically designed to serve medical physics community with pre-built components. The BEAMnrc codes were freely downloaded from National Research Council Canada (at http://www.nrc-cnrc.gc.ca/eng/solutions/advisory/egsnrc_index.html).

First, we modeled an electron beam treatment head according to the linac geometry provided by the manufacturer. To calculate dose distributions in a water phantom, DOSXYZnrc code was used. After that, benchmarking of the modeled treatment head was performed by matching the calculated beam data using DOSXYZnrc with the measured beam data acquired with an ionization chamber in a water phantom in order to obtain realistic MC simulations. After benchmarking, the scattering foil was removed to simulate SFF electron beams. Percent depth doses (PDD) as well as off-axis beam profiles of both the SF and SFF electron beams were acquired for comparison purposes.

**Fig 1:**
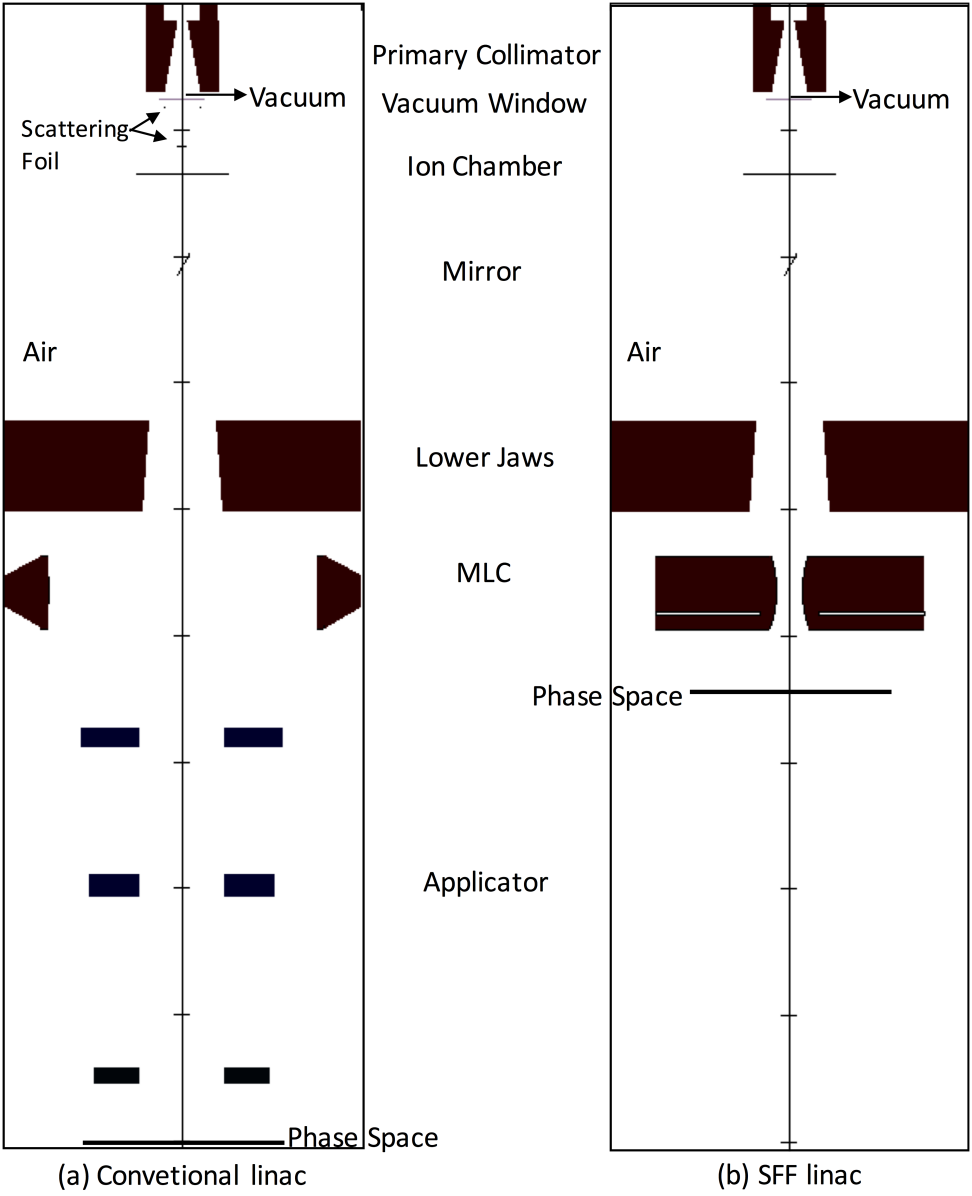
A Schematic diagram of the simulated linac treatment head modeled with BEAMnrc Monte Carlo code. It is shown a conventional linac with (a) and without (b) applicator and scattering foil. (SFF: Scattering Foil Free).

Treatment heads for 6, 9, 12, 16 and 20 MeV electron beams of a Clinac iX^™^ (Varian Medical Systems, Palo Alto, CA, USA) were modeled with pre-defined component modules of BEAMnrc code. Each treatment head included scattering foil, primary collimator, vacuum window, monitoring ion chamber, mirror, secondary collimators, MLCs (Millennium 120 MLC^™^, Varian Medical Systems, Palo Alto, CA, USA) and an electron cone with a field size of 10 × 10 cm^2^ (Fig 1(a)). The field sizes of the secondary collimators were set to be identical those defined by the manufacturer for the 10 × 10 cm^2^ electron cone for each energy. Geometry and material composition of each component was determined according to data provided by the manufacturer. The cutoff energies of photons (PCUT) and electrons (ECUT) for terminating their transport were set to 10 keV and 700 keV, respectively. The PRESTA-II electron transport algorithm was used. Boundary crossing algorithm was set to EXACT. No variance reduction techniques were applied, and the number of simulated histories was 5×10^8^. The electron pencil beam passes through the head of the accelerator. The electron source was a divergent beam with a 2D Gaussian spatial distribution (ISOURC = 19). The full width at half maximum (FWHM) was 1.3 mm in both *x* and *y* directions. The kinetic energies of each electron beam were fine tuned to match the MC simulation with the measured dose distribution. The scattering foils with various sizes were modeled for each electron energy based on the geometric configuration supplied by the manufacturer. The phase space files scoring information about the type, charge, positions, directions, and energies of each particle were generated at the plane orthogonal to the beam direction at 100 cm distance from the source.

The phase space recorded previously in BEAMnrc was used as a source in the DOSXYZnrc code. To calculate PDDs and profiles, a water phantom with dimensions of 20 × 20 × 15 cm^3^ was placed at an SSD of 100 cm. The voxel dimensions of the water phantom were 5 × 5 × 2 mm^3^. Dose deposition simulations were made with same cut-off values in treatment head (ECUT = 700 keV and PCUT = 10 keV). Boundary crossing algorithm was set to PRESTA-I in the DOSXYZnrc simulation. The phase space file was used several times for 3×10^8^ number of histories to keep the statistical uncertainty in PDDs under 2%. For profiles, the statistical uncertainty was kept less than 2% in the region of doses larger than 2%. Benchmarking of the modeling was performed with beam data measured using a CC13 scanning ionization chamber and Blue Phantom^™^ (IBA Dosimetry, Schwarzenbruck, Germany).

### Simulation of SFF electron beams defined with photon MLCs

After benchmarking, following previous studies [7, 8] we removed the electron cone and generated electron beams with a field size of 2 × 2 cm^2^, which was defined with MLCs (2 × 2 cm^2^ was the size at the surface of 100 cm SSD). The size of the secondary collimator was fixed at 20 × 20 cm^2^. We acquired PDDs and profiles of electron beams at 60 cm SSD to acquire sharp penumbrae with MLCs [8]. After that, we removed both upper and lower scattering foils and acquired PDDs and profiles using identical methodology (Fig 1(b)).

The differences between SF and SFF electron beams in the PDDs, as well as off-axis beam profiles at the reference depths were analyzed. In the PDDs, the changes in bremsstrahlung contamination were investigated. The dose increase along the central axis from removal of the scattering foil was also investigated.

### Scanning with SFF electron beams

Cylindrical and spherical virtual water phantoms of various radii were generated. A total of 4 cylindrical water phantoms with radii of 5 cm (Cyl_R5_), 7.5 cm (Cyl_R7.5_), 10 cm (Cyl_R10_) and 15 cm (Cyl_R15_) were generated, each with longitudinal length of 10 cm. The spherical phantoms’ radii were 5 cm (Sph_R5_), 7.5 cm (Sph_R7.5_), 10 cm (Sph_R10_) and 15 cm (Sph_R15_). For both cylindrical and spherical phantoms, the target volumes were defined from the surface to 5 mm depth to simulate skin cancer. To treat these target volumes, 6 MeV SFF electron beam with a 1 cm bolus around the virtual water phantoms were used.

We acquired 3D dose distributions with a single SFF electron beam with a small field size for each phantom. The SFF electron beam was incident perpendicular to the phantom surface and the SSD was 60 cm. A total of 3 types of SFF electron beams were simulated, of which field sizes were 1 × 1 cm^2^, 2 × 2 cm^2^ and 4 × 4 cm^2^. After that, to simulate scanning, the dose distributions calculated with MC were imported to MATLAB (version 8.1, Mathworks Inc., Natick, MA, USA). Dose distributions in virtual phantoms were acquired by superposition of MC calculated dose. For each cylindrical phantom, dose distributions with scanning at intervals of 1°, 2°, 3°, 4°, 5°, 10°, 15° and 20° were investigated. The scanning intervals in the direction of cylindrical phantom length were 0.3 cm, 0.6 cm, 1 cm, 1.3 cm, 1.6 cm and 2 cm. For the spherical phantoms, dose distributions with scanning at intervals of 1°, 2°, 3°, 4°, 5°, 10°, 15° and 20° were calculated. The calculation grids of both cylindrical and spherical phantoms were 2.5 × 2.5 × 2 mm^3^.

### SFF electron beam scanning vs. VMAT

To test the performance of the SFF electron beam scanning technique, we compared dose distributions in the virtual phantoms produced using the SFF scanning technique against those produced with the VMAT technique. The prescription dose was 30 Gy with a daily dose of 3 Gy (10 fractions). Each plan was normalized such that at least 95% volume of the target volume was covered by 95% of the prescription dose. For VMAT planning, the Eclipse^™^ system (version 10, Varian Medical Systems, Palo Alto, CA, USA) was used. The isocenter was located at the centroid of the virtual phantoms. Two full arcs and a 6 MV photon beam were used. For optimization and dose calculation, progressive resolution optimizer 3 (PRO3) and anisotropic analytic algorithm (AAA) were used, respectively (Varian Medical Systems, Palo Alto, CA, USA). The size of the dose calculation grid was 2.5 mm. For both SFF scanning technique and VMAT, dose-volumetric parameters were calculated and compared. For the target volume, the *conformity index* (*CI*) and *homogeneity index* (*HI*) were acquired, which were calculated as follows [20].

$$Conformity\;index\;\left(CI\right)=\;\frac{Volume\;within\;\;95\%\;\;of\;prescription\;dose}{Volume\;of\;target\;volume}$$

$$Homogeneity\;index\;\left(HI\right)=\;\frac{D_{5\%}}{D_{95\%}}$$

where D_5%_ and D_95%_ are the dose received by at least 5% and 95% volume of the target volume, respectively. For normal tissue, mean dose to the body structure was calculated by subtracting the target volume and bolus from whole volume of the phantom.

## Results

### Benchmarking of the MC modeling of the treatment head

**Fig 2:**
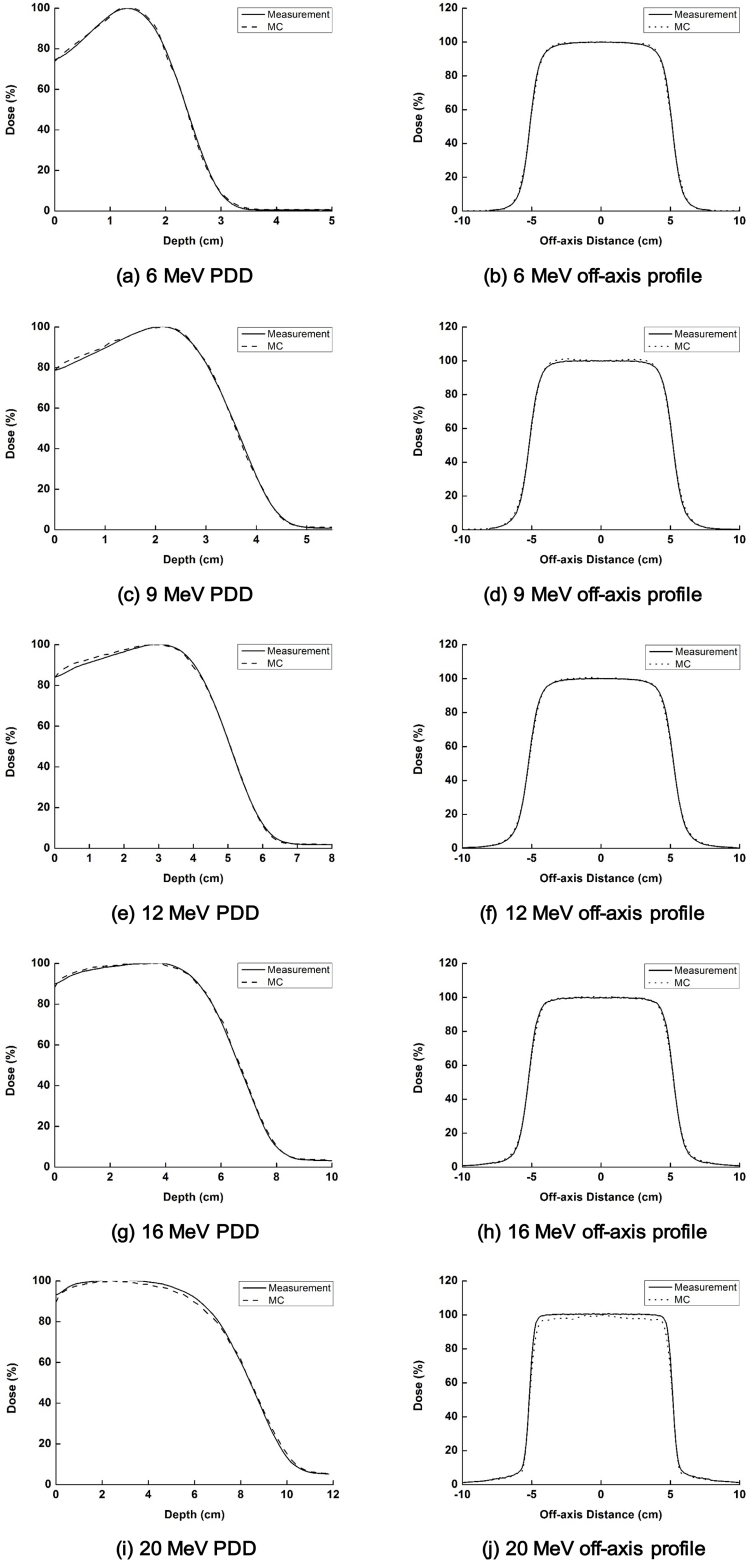
The measured (solid line) and calculated with Monte Carlo (MC) simulation (dashed line) percent depth doses (PDDs) as well as off-axis beam profiles at the reference depths are shown for each electron energy. The PDDs of 6 MeV (a), 9 MeV (c) 12 MeV (e), 16 MeV (g) and 20 MeV (i) and the profiles of 6 MeV (b), 9 MeV (d), 12 MeV (f), 16 MeV (h) and 20 MeV (j) are shown.

The measured and simulated PDDs as well as off-axis profiles of each electron beam (6, 9, 12, 16 and 20 MeV) with a 10 × 10 cm^2^ cone at 100 cm SSD are shown in Fig 2. Profiles at the reference depths according to the AAPM TG-51 protocol are also shown [21]. Good agreement of within 2%/1 mm was obtained for all PDD and profiles to the measurements except the 20 MeV off-axis beam profile which was matched to within 3%/2 mm (S1 Table) [7, 13].

### Beam data of SFF vs. SF electron beams

**Fig 3:**
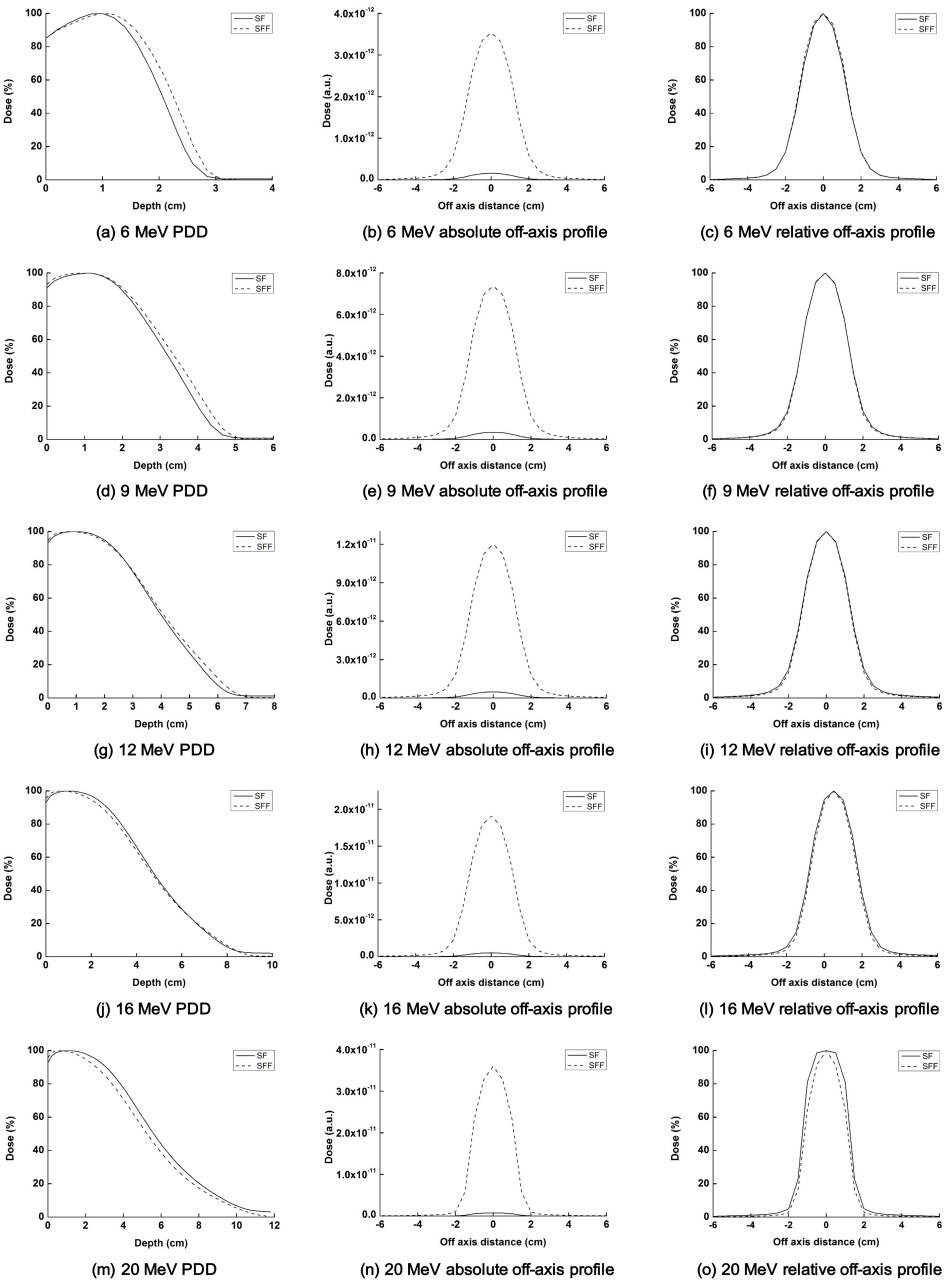
The percent depth doses (PDDs) as well as absolute and normalized off-axis beam profiles at the reference depths of electron beams with scattering foil (solid line) and scattering foil free (dashed line) are shown for each energy of electron beams. The PDDs of 6 MeV (a), 9 MeV (d), 12 MeV (g), 16 MeV (j) and 20 MeV (m) are shown. The absolute off-axis beam profiles of 6 MeV (b), 9 MeV (e), 12 MeV (h), 16 MeV (k) and 20 MeV (n) and relative off-axis beam profiles, i.e. normalized at the value of central axis, of 6 MeV (c), 9 MeV (f), 12 MeV (i), 16 MeV (l) and 20 MeV (o) are shown.

The simulated PDDs as well as off-axis profiles of each electron beam with and without a scattering foil with a field size of 2 × 2 cm^2^ as defined by Millennium 120^™^ MLCs at 60 cm SSD are shown in Fig 3 (S2–S5 Tables). The changes in beam characteristics from removing scattering foil from the electron beam path are summarized in Table 1. The output of 6, 9, 12, 16 and 20 MeV SFF beams increased by 21, 21, 25, 37 and 51 times, respectively, in comparison with those of the SF beams. The output increase of high energy electron beams (20 MeV) from removal of the scattering foil was much larger than for low energy (6 MeV) electron beams (51 times vs. 21 times). Since the field sizes were small (2 × 2 cm^2^) and the electron cone was removed, the off-axis profile of the SFF beam was similar with that of SF beam in shape. In the cases of 6, 9 and 12 MeV, the values of R_50_ of SFF beams were higher than those of SF beams, while the R_50_ of SFF beams were lower than those of SF beams for 16 and 20 MeV beams. As shown by Eldib *et al*., the bremsstrahlung contamination of SFF beams was much lower than SF beams for all energy levels investigated in this study [13].

**Table 1:** Beam characteristics of scattering foil free electron beams.

	Output increase (SFF^a^/SF^b^)	SF R_50_ (cm)	SFF R_50_ (cm)	SF photon contamination (%)	SFF photon contamination (%)
6 MeV	20.88	2.0	2.2	0.49	0.07
9 MeV	21.44	3.2	3.4	0.82	0.20
12 MeV	25.18	4.1	4.2	1.15	0.21
16 MeV	37.41	4.8	4.6	1.98	0.25
20 MeV	50.68	5.7	5.3	3.08	0.42

^a^ Scattering foil free electron beam with a field size of 2 cm × 2 cm collimated with multi-leaf collimator

^b^ Scattering foil electron beam with a field size of 2 cm × 2 cm collimated with multi-leaf collimator

### Optimal scanning parameters for a cylindrical phantom

**Fig 4:**
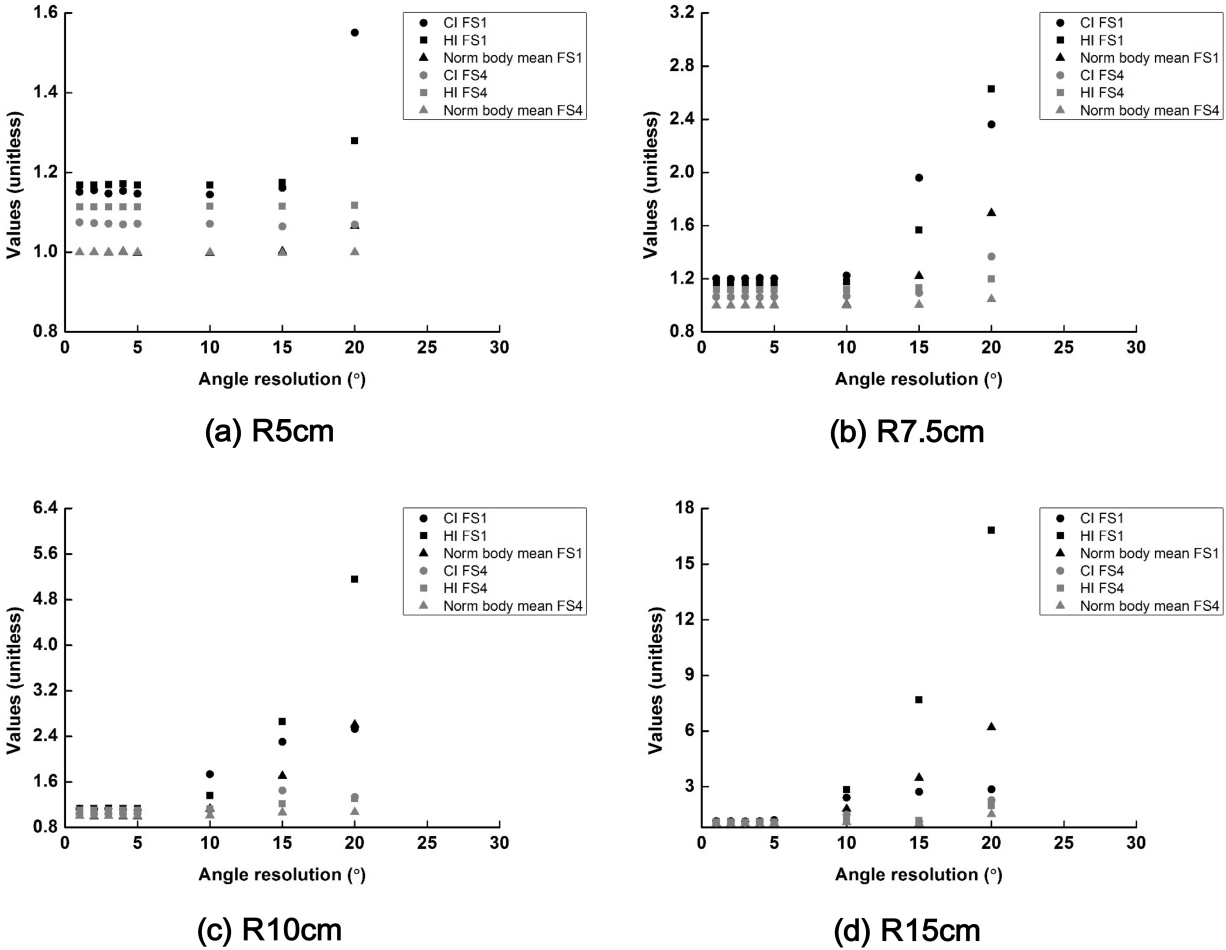
The changes in the values of conformity index (CI), homogeneity index (HI) and normalized body mean dose (body mean dose with a certain scanning angle resolution/body mean dose with a scanning angle resolution of 1°) of the scattering filter free (SFF) scanning electron beam plans with field sizes of 1 cm × 1 cm and 4 cm × 4 cm are plotted according to the various scanning angle resolutions. Those dose-volumetric parameters acquired with cylindrical phantoms with radii of 5 cm (a), 7.5 cm (b), 10 cm (c) and 15 cm (d) are shown. The CI, HI and normalized body mean dose are plotted with circle, square and triangle, respectively. The values with a field size of 1 cm × 1 cm are shown in black color while those with a field size of 4 cm × 4 cm are shown in gray color.

The changes in the values of *CI*, *HI* and normalized body mean dose (body mean dose with a certain scanning angle resolution/body mean dose with a scanning angle resolution of 1°) of the SFF scanning beams with field sizes of 1 × 1 cm^2^ and 4 × 4 cm^2^ are plotted according to the scanning angle resolutions in Fig 4. The scanning resolution along the longitudinal direction was 3 mm. When the scanning angle resolution resulted in a larger beam separation than the field size, the *CI* and *HI* indicated poor plan quality for the target volume because the SFF beams were not fully overlapped due to the large beam separation. Moreover, because the target coverage was normalized to cover 95% of the target volume by 95% of the prescription dose, body mean doses were also increased when the SFF beams were not fully overlapped. The plan quality of scanning beams with a field size of 4 cm × 4 cm changed minimally according to the scanning angle resolution up to 20° because the scanning beams were sufficiently overlapped up to 20° scanning angle resolution.

**Fig 5:**
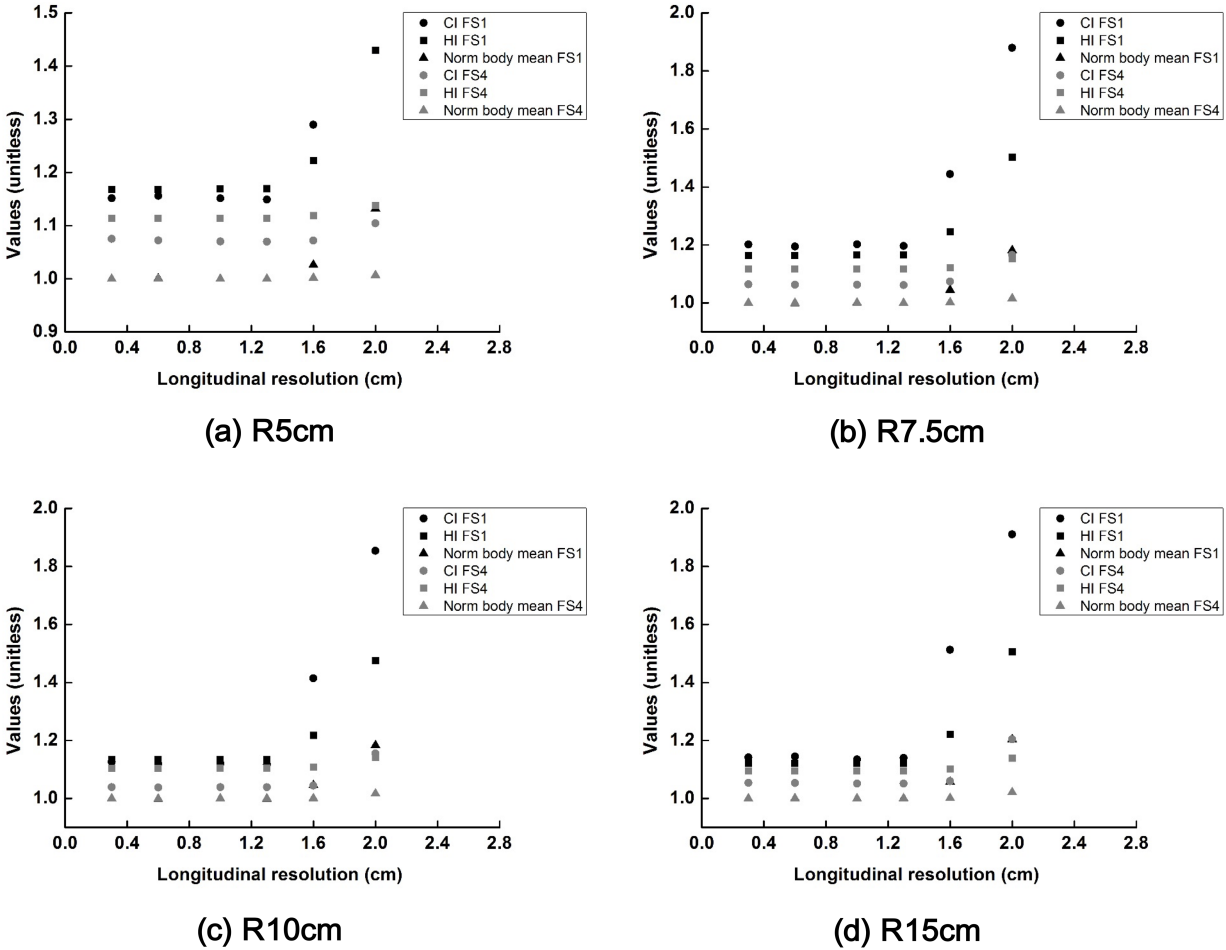
The changes in the values of conformity index (CI), homogeneity index (HI) and normalized body mean dose (body mean dose with a certain longitudinal scanning resolution/body mean dose with a longitudinal scanning resolution of 3 mm) of the scattering filter free (SFF) scanning electron beam plans with field sizes of 1 cm × 1 cm and 4 cm × 4 cm are plotted according to the various longitudinal scanning resolutions. Those dose-volumetric parameters acquired with cylindrical phantoms with radii of 5 cm (a), 7.5 cm (b), 10 cm (c) and 15 cm (d) are shown. The CI, HI and normalized body mean dose are plotted with circle, square and triangle, respectively. The values with a field size of 1 cm × 1 cm are shown in black color while those with a field size of 4 cm × 4 cm are shown in gray color.

The changes in values of *CI*, *HI* and normalized body mean dose (body mean dose with a certain longitudinal resolution/body mean dose with a longitudinal resolution of 3 mm) of the scanning beams with field sizes of 1 × 1 cm^2^ and 4 × 4 cm^2^ are plotted according to the longitudinal resolutions in Fig 5. The scanning angle resolution was fixed to be 1°. In the case of the scanning beam with a field size of 1 × 1 cm^2^, when the longitudinal resolution was 1.6 cm, the plan quality was drastically reduced as the scanning beams were not sufficiently overlapped. That poor plan quality was not observed for the scanning beam with a field size of 4 × 4 cm^2^ because the maximum beam separation along the longitudinal direction was only 2 cm in this study.

In the case of a cylindrical phantom, if the scanning resolution was larger than the field size of the scanning beam, the plan quality was poor for both the target volume and normal tissue.

### Optimal scanning parameters for a spherical phantom

**Fig 6:**
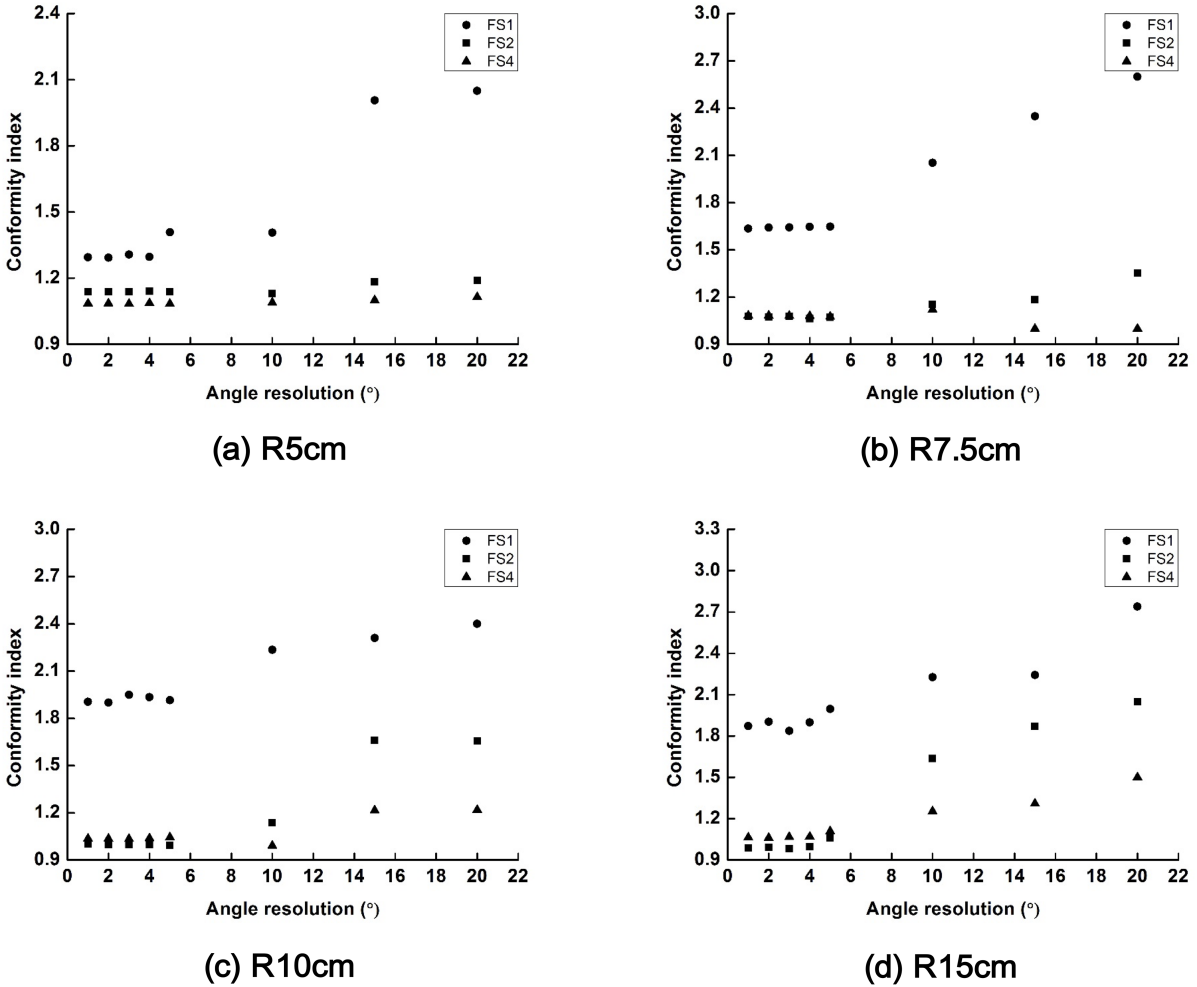
The changes in the values of conformity index (CI) of the scattering filter free (SFF) scanning electron beam plans with field sizes of 1 cm × 1 cm, 2 cm × 2 cm and 4 cm × 4 cm are plotted according to the various scanning angle resolutions. Those CIs acquired with spherical phantoms with radii of 5 cm (a), 7.5 cm (b), 10 cm (c) and 15 cm (d) are shown. The CI with a field size of 1 cm × 1 cm, 2 cm × 2 cm and 4 cm × 4 cm are plotted with circle, square and triangle, respectively.

The changes in the values of *CI* of the scanning beams with field sizes of 1 × 1 cm^2^, 2 × 2 cm^2^ and 4 × 4 cm^2^ are plotted according to the scanning angle resolution in Fig 6. Similar to the results of the cylindrical phantom, the target conformity was worsened when the scanning angle resolution resulted in a larger separation between beams than the field size of the scanning beam. In general, the target conformity of the scanning beams with a large field size (4 × 4 cm^2^) was better than the others. The target conformity of the scanning beams with a field size of 4 × 4 cm^2^ in the Sph_R5_ and Sph_R7.5_ were changed minimally by the scanning angle resolution from 1° to 20°. However, those values were changed considerably in the Sph_R10_ and Sph_R15_ when the scanning angle resolution was large.

**Fig 7:**
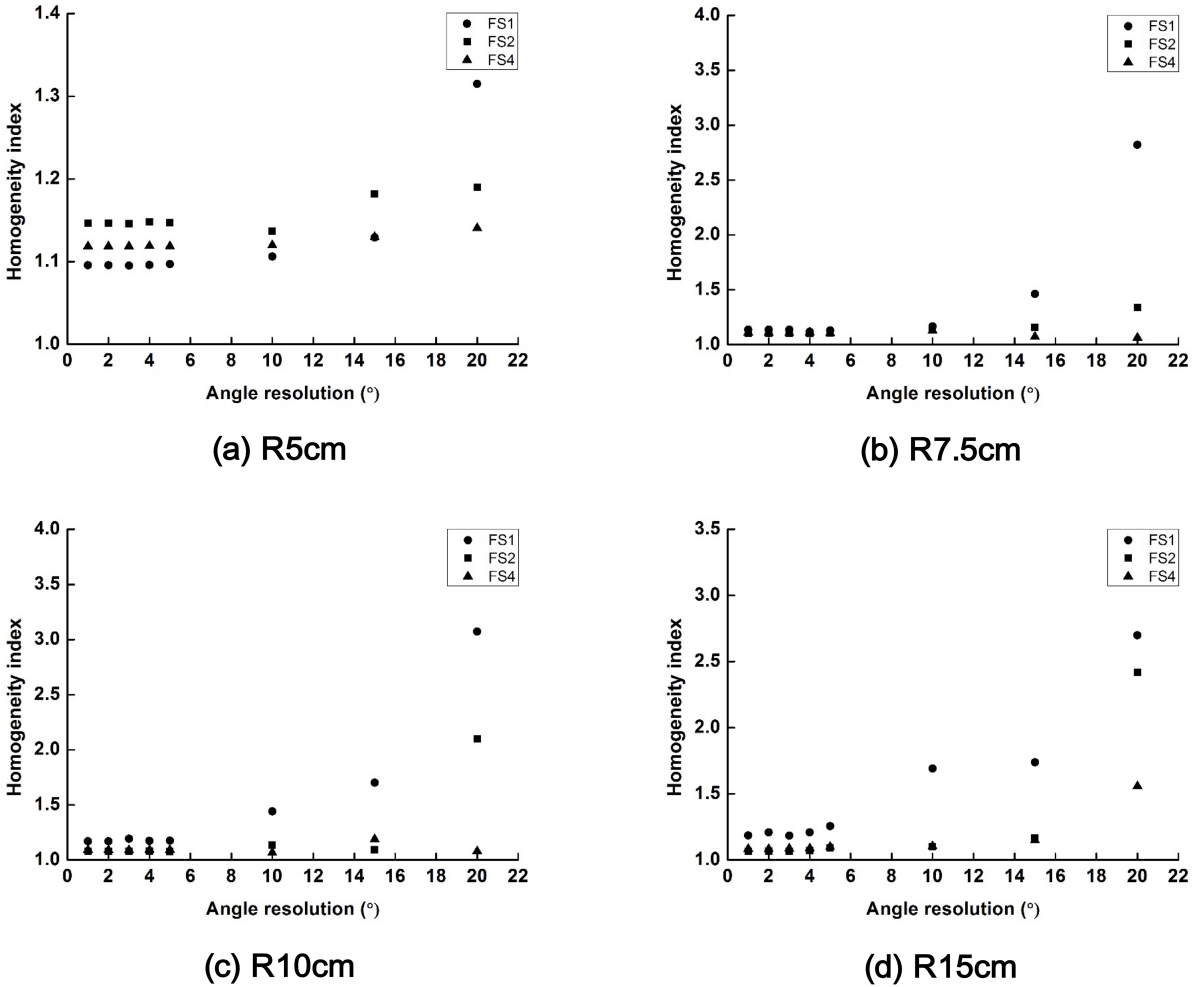
The changes in the values of homogeneity index (HI) of the scattering filter free (SFF) scanning electron beam plans with field sizes of 1 cm × 1 cm, 2 cm × 2 cm and 4 cm × 4 cm are plotted according to the various scanning angle resolutions. Those HIs acquired with spherical phantoms with radii of 5 cm (a), 7.5 cm (b), 10 cm (c) and 15 cm (d) are shown. The HI with a field size of 1 cm × 1 cm, 2 cm × 2 cm and 4 cm × 4 cm are plotted with circle, square and triangle, respectively.

The changes in the values of *HI* of the scanning beams with field sizes of 1 × 1 cm^2^, 2 × 2 cm^2^ and 4 × 4 cm^2^ are plotted according to the scanning angle resolution in Fig 7. The dose homogeneity in the target volume was worsened when the scanning angle resolution resulted in a larger separation between beams than the field size of the scanning beam. In the case of SPh_R5_, target homogeneity with a field size of 1 × 1 cm^2^ was better than the others when the scanning angle resolution was small. However, for large phantoms, target homogeneity with a field size of 1 × 1 cm^2^ was worse than the others. The scanning beam with a field size of 4 cm × 4 cm showed minimal changes in the target homogeneity as the scanning angle resolution was varied from 1° to 20°.

**Fig 8:**
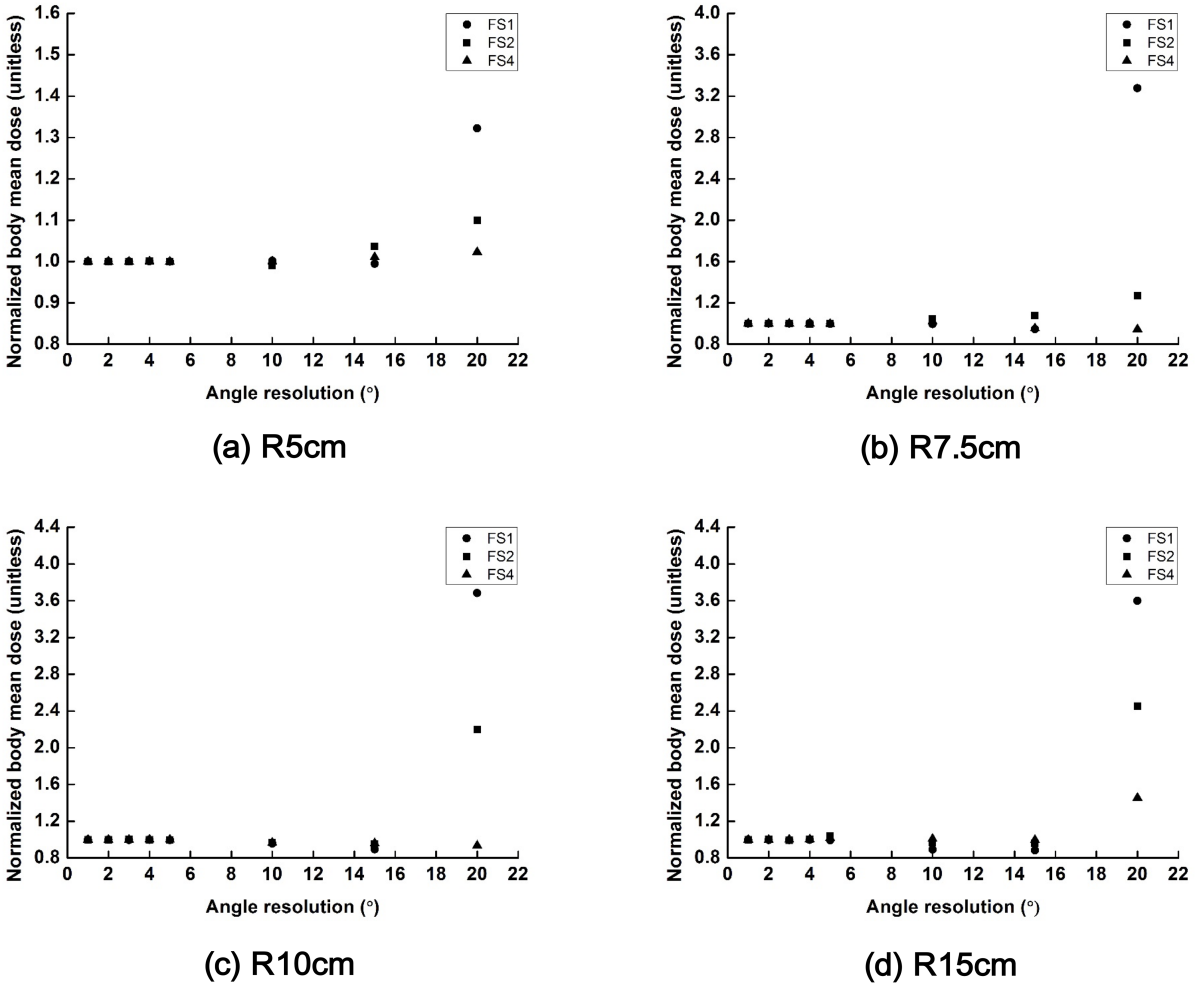
The changes in the values of normalized body mean dose (body mean dose with a certain scanning angle resolution/body mean dose with a scanning angle resolution of 1°) of the scattering filter free (SFF) scanning electron beam plans with field sizes of 1 cm × 1 cm, 2 cm × 2 cm and 4 cm × 4 cm are plotted according to the various scanning angle resolutions. Those normalized body mean doses acquired with spherical phantoms with radii of 5 cm (a), 7.5 cm (b), 10 cm (c) and 15 cm (d) are shown. The normalized body mean doses with a field size of 1 cm × 1 cm, 2 cm × 2 cm and 4 cm × 4 cm are plotted with circle, square and triangle, respectively.

The changes in the values of the normalized body mean dose (body mean dose with a certain scanning angle resolution/body mean dose with a scanning angle resolution of 1°) of the scanning beams with field sizes of 1 × 1 cm^2^, 2 × 2 cm^2^ and 4 × 4 cm^2^ are plotted according to the scanning angle resolution in Fig 8. The normal tissue irradiation increased when the scanning angle resolution resulted in larger separation between beams than the field size of the scanning beam especially for the scanning angle of 20°. The scanning beam with a field size of 4 × 4 cm^2^ showed minimal changes in the body mean dose as the scanning angle resolution was varied from 1° to 20° in all spherical phantoms except the Sph_R15_. In the SPh_R15_, body mean dose of the scanning beam with a field size of 4 × 4 cm^2^ and scanning angle resolution of 20° increased significantly.

### SFF electron beam technique vs. VMAT for the skin cancer on cylindrical surface

**Fig 9:**
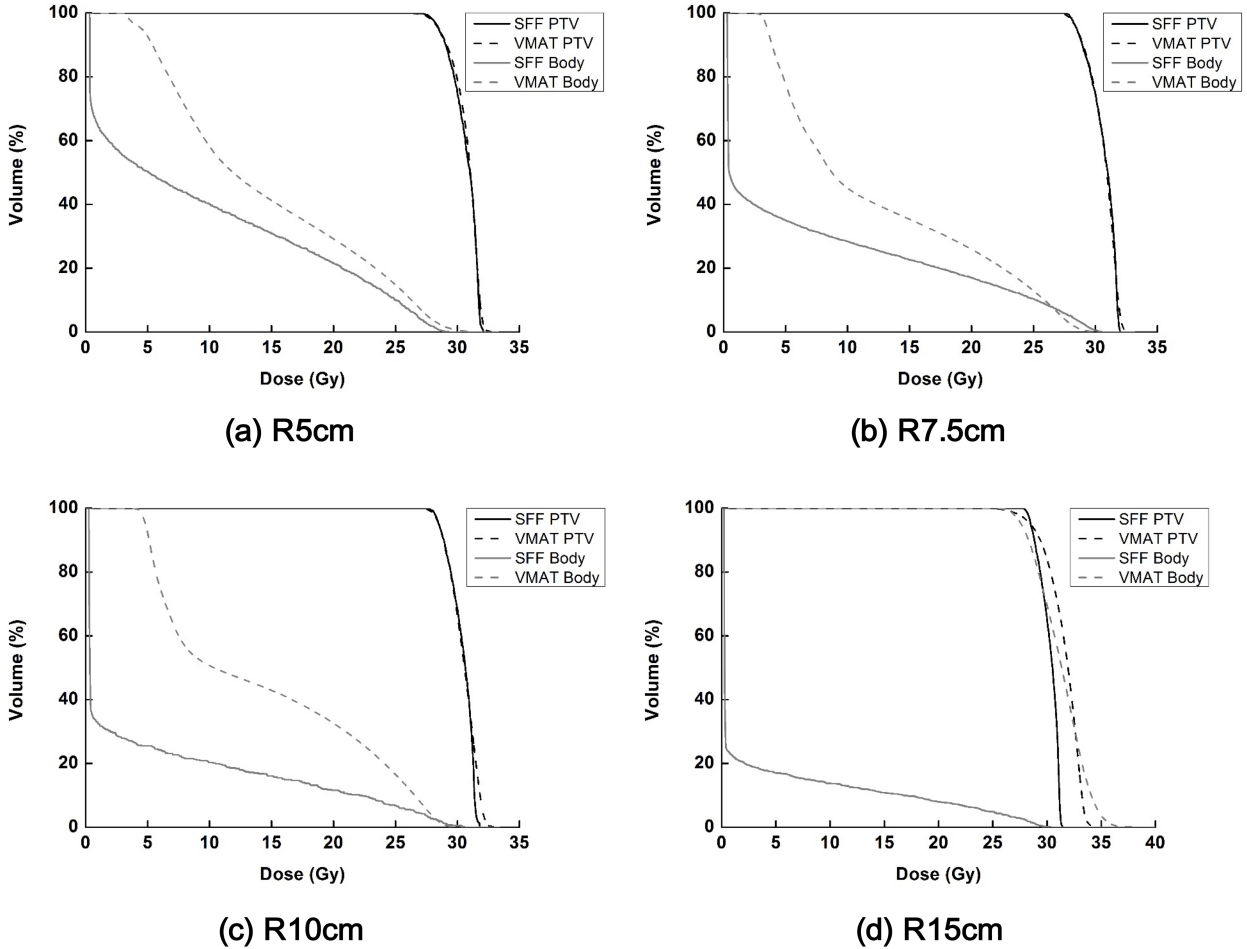
The dose volume histograms (DVHs) of the target volumes as well as normal tissues (body structure–target volume) calculated from cylindrical phantoms are shown in black and gray color, respectively. The DVHs acquired with the cylindrical phantoms with radii of 5 cm (a), 7.5 cm (b), 10 cm (c) and 15 cm (d) are shown. The DVHs of the scattering foil free (SFF) scanning plans and volumetric modulated arc therapy plans (VMAT) are shown in solid and dashed lines, respectively.

**Fig 10:**
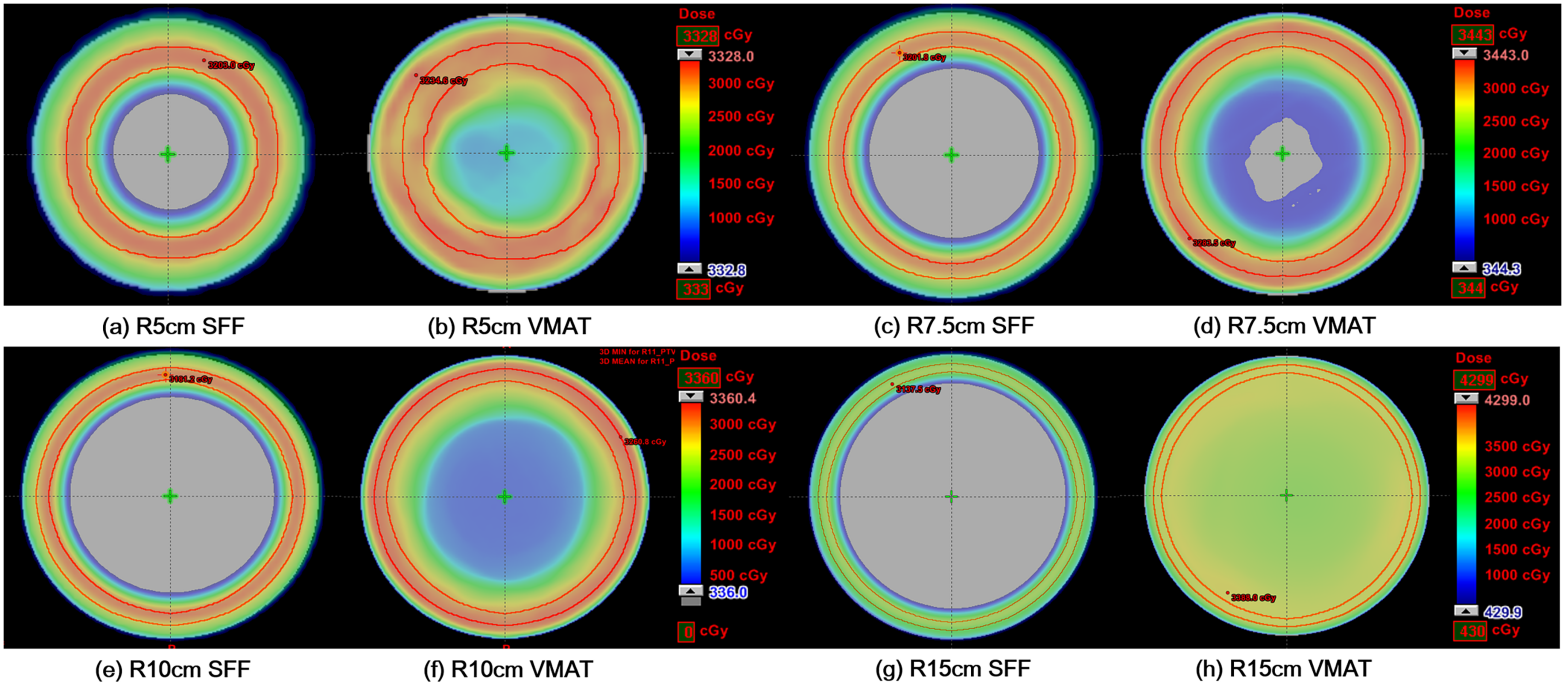
The dose distributions in the axial cut calculated with cylindrical phantoms with radii of 5 cm (a), 7.5 cm (b), 10 cm (c) and 15 cm (d) of the scattering filter free (SFF) electron beam scanning plans and volumetric modulated arc therapy (VMAT) plans are shown. The dose distributions of SFF plans acquired with the cylindrical phantoms with radii of 5 cm (a), 7.5 cm (c), 10 cm (e) and 15 cm (g) are shown. The dose distributions of VMAT plans acquired with the cylindrical phantoms with radii of 5 cm (b), 7.5 cm (d), 10 cm (f) and 15 cm (h) are also shown.

Dose volume histograms (DVH) of the target volumes and normal tissues calculated from the SFF beam plans and VMAT plans in the Cyl_R5_, Cyl_R7.5_, Cyl_R10_ and Cyl_R15_ are shown in Fig 9. The dose distributions in the axial image of the Cyl_R5_, Cyl_R7.5_, Cyl_R10_ and Cyl_R15_ with the SFF and VMAT technique are shown in Fig 10. The SFF plans were generated with a field size of 4 × 4 cm^2^, scanning angle resolution of 1° and longitudinal resolution of 3 mm (the finest scanning resolution). The dose-volumetric parameters calculated from SFF beam plans and VMAT plans for each cylindrical phantom are summarized in Table 2. As shown in Fig 9, the SFF scanning technique reduced dose to normal tissue considerably, particularly when the target volume was large. Keeping the same target coverage, the body mean doses in the Cyl_R5_, Cyl_R7.5_, Cyl_R10_ and Cyl_R15_ of the SFF scanning plans were reduced by 52.9%, 77.8%, 176.7% and 786.7%, respectively, in comparison with those of VMAT plans. The target conformity of the VMAT plans in the Cyl_R5_, Cyl_R7.5_, Cyl_R10_ and Cyl_R15_ were improved by using the SFF scanning technique by 28.0%, 44.3%, 48.1% and 561.9%, respectively, compared to VMAT. The VMAT plan of the Cyl_R15_ was extremely poor due to the leaf span of the Millennium 120^™^ MLC which is 14.5 cm, which resulted in inappropriate modulation.

**Table 2:** Dose-volumetric parameters of scattering foil free scanning technique with 6 MeV electrons and volumetric modulated arc therapy in cylindrical and spherical phantoms with various radii.

	R_5cm_ SFF^a^	R_5cm_ VMAT^b^	R_7.5cm_ SFF	R_7.5cm_ VMAT	R_10cm_ SFF	R_10cm_ VMAT	R_15cm_ SFF	R_15cm_ VMAT
Cylindrical phantom								
Conformity index	1.07	1.37	1.06	1.53	1.04	1.54	1.05	6.95
Homogeneity index	1.12	1.12	1.12	1.12	1.10	1.12	1.10	1.17
Body mean dose (Gy)	9.35	14.30	7.03	12.50	5.06	14.00	3.53	31.30
Spherical phantom								
Conformity index	1.02	1.29	1.00	1.19	1.04	1.83	1.00	3.15
Homogeneity index	1.08	1.09	1.06	1.07	1.08	1.09	1.07	1.07
Body mean dose (Gy)	14.12	11.00	9.31	12.68	7.04	25.55	4.78	28.04

^a^ Treatment plan with scattering foil free electron beams for the treatment of tumor volume ranged from the surface to the depth of 5 mm in a phantom with a radius of 5 cm

^b^ Volumetric modulated arc therapy plan for the treatment of tumor volume ranged from the surface to the depth of 5 mm in a phantom with a radius of 5 cm

### SFF electron beam technique vs. VMAT for the skin cancer on spherical surface

**Fig 11:**
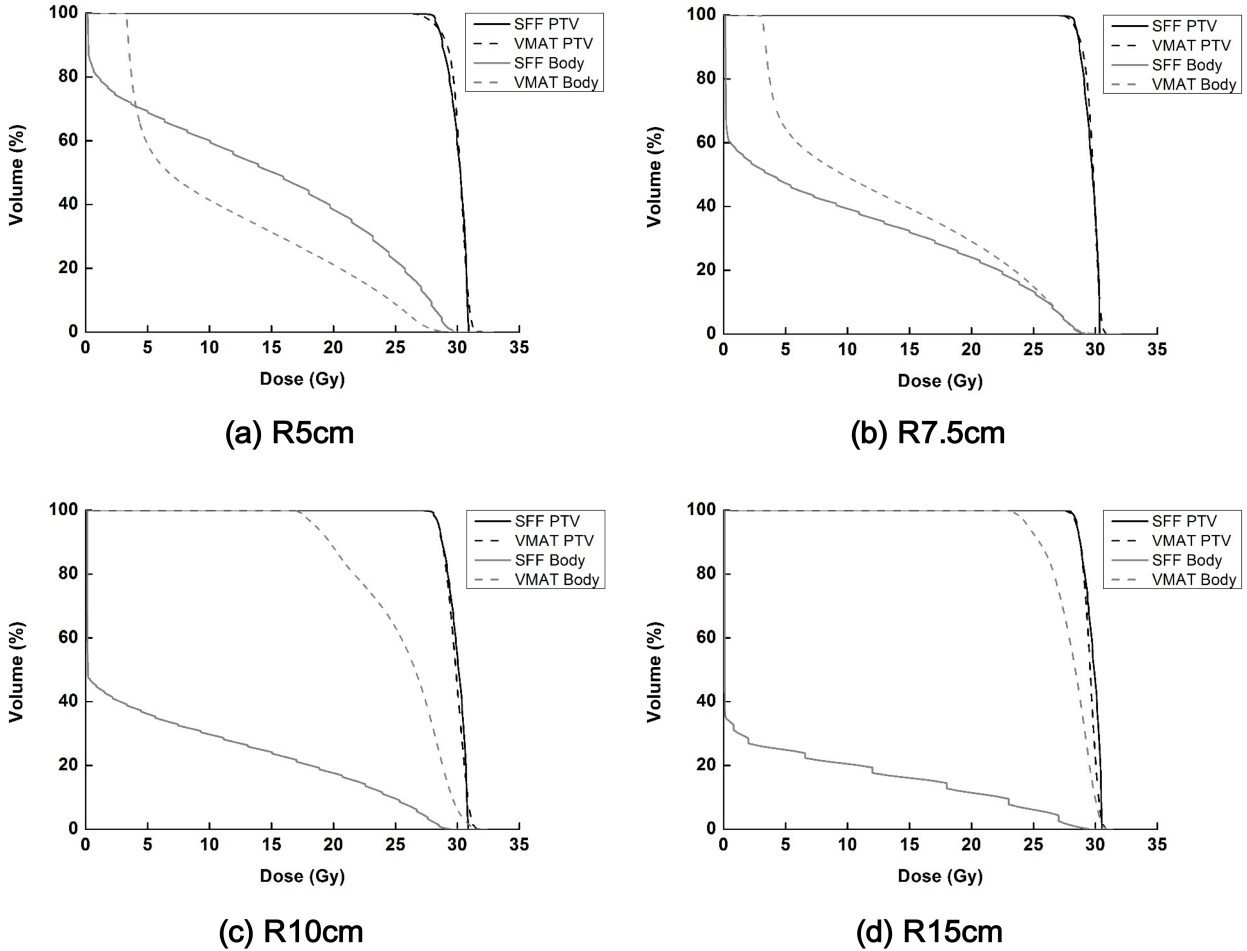
The dose volume histograms (DVHs) of the target volumes as well as normal tissues (body structure–target volume) calculated from spherical phantoms are shown in black and gray color, respectively. The DVHs acquired with the spherical phantoms with radii of 5 cm (a), 7.5 cm (b), 10 cm (c) and 15 cm (d) are shown. The DVHs of the scattering foil free (SFF) scanning plans and volumetric modulated arc therapy plans (VMAT) are shown in solid and dashed lines, respectively.

**Fig 12:**
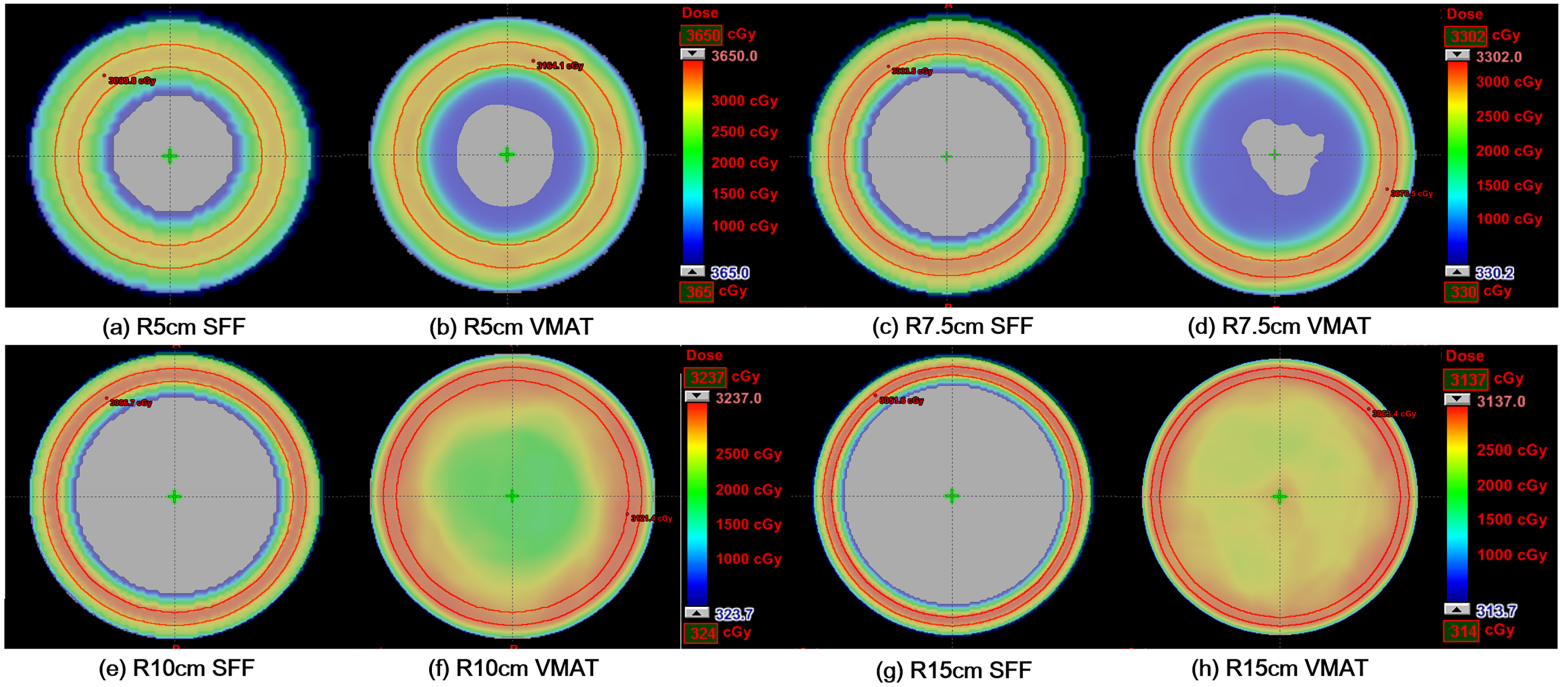
The dose distributions in the axial cut calculated with spherical phantoms with radii of 5 cm (a), 7.5 cm (b), 10 cm (c) and 15 cm (d) of the scattering filter free (SFF) electron beam scanning plans and volumetric modulated arc therapy (VMAT) plans are shown. The dose distributions of SFF plans acquired with the spherical phantoms with radii of 5 cm (a), 7.5 cm (c), 10 cm (e) and 15 cm (g) are shown. The dose distributions of VMAT plans acquired with the cylindrical phantoms with radii of 5 cm (b), 7.5 cm (d), 10 cm (f) and 15 cm (h) are also shown.

Dose volume histograms (DVH) of the target volumes as well as normal tissues calculated from the SFF beam plans and VMAT plans in the Sph_R5_, Sph_R7.5_, Sph_R10_ and Sph_R15_ are shown in Fig 11. The dose distributions in the axial image of the Sph_R5_, Sph_R7.5_, Sph_R10_ and Sph_R15_ with the SFF and VMAT technique are shown in Fig 12. The SFF plans were generated with a field size of 4 × 4 cm^2^ and scan angle resolution of 1° (the finest scanning resolution). The dose-volumetric parameters calculated from SFF beam plans and VMAT plans for each spherical phantom are summarized in Table 2. Similar to the results of the cylindrical phantoms, SFF scanning technique reduced dose to normal tissues considerably, with the exception of the Sph_R5_. In that case, the body mean dose of the SFF plan was larger than the VMAT plan by 22.1%. As the phantom size increased, the degree of sparing of normal tissue increased. Keeping the same target coverage, the body mean doses of the SFF scanning plans in the Sph_R7.5_, Sph_R10_ and Sph_R15_ were reduced by 36.2%, 262.9% and 486.6%, respectively, in comparison with those of VMAT plans. The target conformity of the VMAT plans in the Sph_R5_, Sph_R7.5_, Sph_R10_ and Sph_R15_ were improved by using the SFF scanning technique by 26.5%, 19.0%, 76.0% and 215.0%, respectively. The VMAT plan of the Sph_R15_ was extremely poor for the same MLC leaf span reasons stated above for the cylindrical.

## Discussion

Currently, there is no optimal solution for the treatment of extensively developed skin cancer on the scalp or other irregular patient surfaces because the conventional electron beam therapy is limited in application to skin cancer on irregular or large patient surfaces [3, 6, 22]. Therefore, several studies have suggested HDR brachytherapy with a surface applicator, IMRT or VMAT as alternative to electron beam therapy to treat skin cancer on the scalp or extremities [3, 6, 22–24]. The photon-electron combined technique was even developed to improve dose distribution on scalp [25]. However, those techniques still involve considerable normal tissue irradiation due to inherited characteristics of high penetrating power of the photon beam. Therefore, as a potential treatment method for skin cancer developed on large or irregular patient surfaces, we proposed a new treatment technique: SFF electron beam scanning therapy, and investigated its performance as compared to the VMAT technique with MC simulation in this study. Although the proposed technique is not available currently, this treatment technique seems feasible technically because the scanning technique of the incident electron beam using the magnet in the treatment head (MM50 racetrack microtron) as well as synchronized movements of couch, dose-rate and gantry rotation (developer mode of TrueBeam) are already available technically [17, 18]. Before proposing a new treatment technique, its benefit compared to the conventional technique should be identified. Therefore, we demonstrated the dosimetric superiority of the proposed technique for the treatment of skin cancer located on the scalp as compared to the VMAT technique as a proof of concept study. The result showed that the proposed technique could drastically reduce dose to normal tissue in comparison with VMAT for the scalp treatment while maintaining the same target coverage. The SFF electron beam scanning technique reduced body mean dose by 263% compared to VMAT in a spherical phantom with a radius of 10 cm, which is comparable in size to a Caucasian adult male’s head [26]. In that phantom, the value of *CI* of the SFF scanning plan was 1.04 while that of using VMAT was 1.83. The proposed technique has a clear benefit in terms of normal tissue sparing compared to the current state-of-the-art radiotherapy technique, VMAT. When the target volume was large, this benefit of the proposed technique was maximized.

As Connell *et al*. and Eldib *et al*. already demonstrated, benefits of the SFF electron beam, such as low bremsstrahlung contamination and output increase, were clearly shown in this study (Fig 3 and Table 1) [7, 13]. The SFF beam characteristics of low energies (6, 9 and 12 MeV electrons) with a small field size, *i*.*e*. 2 × 2 cm^2^, were similar to those of the study by Eldib *et al*. [13]. For low energy electrons, removing the scattering foil increased PDD after the depth of dose maximum (D_max_), increased output, and significantly reduced bremsstrahlung contamination [13]. Since we used a small field size and the electron cone was removed in this study, the shape of the off-axis beam profiles of SF beams were similar to those of SFF beams (both were not flat). In the cases of high energy electron beams (16 and 20 MeV electrons), the PDD after D_max_ was reduced in SFF beams as compared to SF beams, which was a contradictory result to the results of Eldib *et al*. This might be attributed to the small field size of 2 × 2 cm^2^ in this study. The field size of the study by Eldib *et al*. was larger than 10 × 10 cm^2^ [13]. As electron energy was increased, the range of the scattered electrons by scattering foil increased, which might contribute to the increase in dose along the central axis of the SF beams [5]. Further study on the changes in the SFF beam characteristics compared to the SF beams with various electron energies and field sizes will be performed in the future.

We tried to find optimal parameters for the SFF scanning technique. In the results, the plan quality was good when the scanning resolution was small enough to result in full overlap between the scanning beams. At the very least, the scanning intervals should be smaller than the field size of the scanning beam to ensure good plan quality. If the scanning resolution was larger than the field size of the scanning beam, drastic degradation of the plan quality was observed. If the scanning resolution was fine enough for full overlap between beams, the field size of 4 × 4 cm^2^ generally resulted in a slightly better plan quality than did the beams with a field size of 1 × 1 cm^2^. For both cylindrical and spherical phantoms, target conformity as well as body mean dose with the field size of 4 × 4 cm^2^ were better than those with the field size of 1 × 1 cm^2^ when the scanning angle was equal to or less than 10° for all phantom sizes. Because fine resolution scanning takes a long beam delivery time, the scanning angle of 10° with a field size of 4 × 4 cm^2^ seems optimal considering both plan quality as well as treatment time.

Treatment time for the proposed technique was not calculated in this study. The SFF scanning technique synchronized with gantry rotation and couch movement in order to deliver electron beams perpendicular to the patient surface while maintaining the SSD might result in long treatment times. However, we hypothesize that the increased beam output of the SFF beam may partially counteract the increased treatment time. Although the treatment time of the proposed technique is potentially long, the SFF scanning technique provides value due to its considerable dosimetric advantages in comparison with other radiotherapy techniques for the treatment of extensively developed skin cancer on irregular patient surfaces.

In this study, we used virtual cylindrical and spherical phantoms with radii from 5 cm to 15 cm because the average radius of a 2-months-old female’s head is 5.9 cm and the average radius of Caucasian adult male’s head is 10.08 cm [26]. As shown in Figs 9–12, for patients of any age of either sex, the suggested technique can reduce dose to normal tissue significantly as compared to conventional state-of-the-art techniques (VMAT) for the treatment of skin cancer on the scalp. Although we couldn’t investigate the SFF scanning electron beams with real machines in this study, the advantages of SFF scanning beam technique for the treatment of skin cancer extensively developed on irregular surface were identified. The present data in this study can be used as a rationale for the development of an SFF scanning electron beam machine due to the considerable dosimetric advantage which cannot be currently be achieved by any other commercial radiotherapy system.

## Conclusions

By removing scattering foil in the treatment head of 6 MeV electron beam with a field size of 2 × 2 cm^2^, beam output increased by 21 times and bremsstrahlung contamination decreased by 7 times compared to the 6 MeV electron beam with scattering foil. With MC simulations, we demonstrated that the SFF scanning technique with 6 MeV electrons can reduce mean dose to normal tissue by 260% in a spherical phantom that is comparable to the average size of a Caucasian adult male’s head (10 cm radius) while keeping same target coverage, as compared to the VMAT technique. The target conformity and homogeneity of the SFF scanning technique were also better than those of VMAT.

## Supporting information

S1 Table Monte Carlo benchmarking results of the 6 MeV electron beam. The benchmarking results of the simulated 6 MeV electron beam are shown.
(XLSX)

S2 Table 6 MeV with a field size of 1 cm by 1 cm. The 6 MeV electron beams with a field size of 1 cm by 1 cm, which were collimated by the multi-leaf collimators are shown with and without scattering foil.
(XLSX)

S3 Table 6 MeV with a field size of 2 cm by 2 cm. The 6 MeV electron beams with a field size of 2 cm by 2 cm, which were collimated by the multi-leaf collimators are shown with and without scattering foil.
(XLSX)

S4 Table 6 MeV with a field size of 4 cm by 4 cm. The 6 MeV electron beams with a field size of 4 cm by 4 cm, which were collimated by the multi-leaf collimators are shown with and without scattering foil.
(XLSX)

S5 Table 6 MeV with a field size of 10 cm by 10 cm. The 6 MeV electron beams with a field size of 10 cm by 10 cm, which were collimated by the multi-leaf collimators are shown with and without scattering foil.
(XLSX)
